# Fabrication and characterization of electrically conducting electrochemically synthesized polypyrrole-based enzymatic biofuel cell anode with biocompatible redox mediator vitamin K_3_

**DOI:** 10.1038/s41598-024-53005-3

**Published:** 2024-02-09

**Authors:** Maha Khan, Inamuddin

**Affiliations:** Advanced Functional Materials Laboratory, Department of Applied Chemistry, Zakir Husain College of Engineering and Technology, Faculty of Engineering and Technology, Aligarh Muslim University, Aligarh, 202002 India

**Keywords:** Fuel cells, Energy

## Abstract

Enzymatic biofuel cells (EBFCs) hold tremendous potential to power biomedical devices, biosensors, and bioelectronics. Unlike conventional toxic batteries, these electrochemical devices are biocompatible, harnessing energy from physiological fluids and producing usable electrical energy. But the commercialization of EBFCs is limited by the low operational stability, limited power output and poor electron transport efficiency of the enzymatic electrodes. In this study, a novel bioanode exhibiting a high electron transfer ability and long-term stability was fabricated. For the preparation of the anode, surfactant-assisted polypyrrole (PPy) was electrochemically co-deposited on a platinum wire with the simultaneous entrapment of vitamin K_3_ (VK_3_) and GOx (glucose oxidase) in the PPy matrix. Herein, conducting PPy acts as an electron transfer enhancer and provides appropriate electrical communication between the active site of the enzyme glucose oxidase (GOx) and the electrode surface. Biocompatible redox mediator vitamin K_3_ was employed as an electron transfer mediator to shuttle electrons between the oxidized fuel glucose and surface of the electrode in the electrochemical cell. The electrical conductivity of PPy was measured using the four-probe technique of conductivity measurement of semiconductors. The morphological characterization of as-synthesized anode (PPy/CTAB/VK_3_/GOx) was performed by Fourier transform infrared (FTIR) spectroscopy, thermogravimetric analysis (TGA), X-ray diffraction (XRD), scanning electron microscopy (SEM) and transmission electron microscopy (TEM). The electrochemical characterization was studied by cyclic voltammetry (CV), linear sweep voltammetry (LSV) and electrochemical impedance spectroscopy (EIS) techniques. It was observed that the room-temperature conductivity of PPy lies in the semiconducting range and it also shows good stability on exposure to laboratory air, making it a promising material to provide electrical contact. The study developed a bioanode producing a modest current density of 6.35 mA cm^–2^ in 20 mM glucose solution. The stability, current output and ease of manufacturing process of the electrode make it particularly suitable for employment in biofuel cell applications.

## Introduction

**Figure 1:**
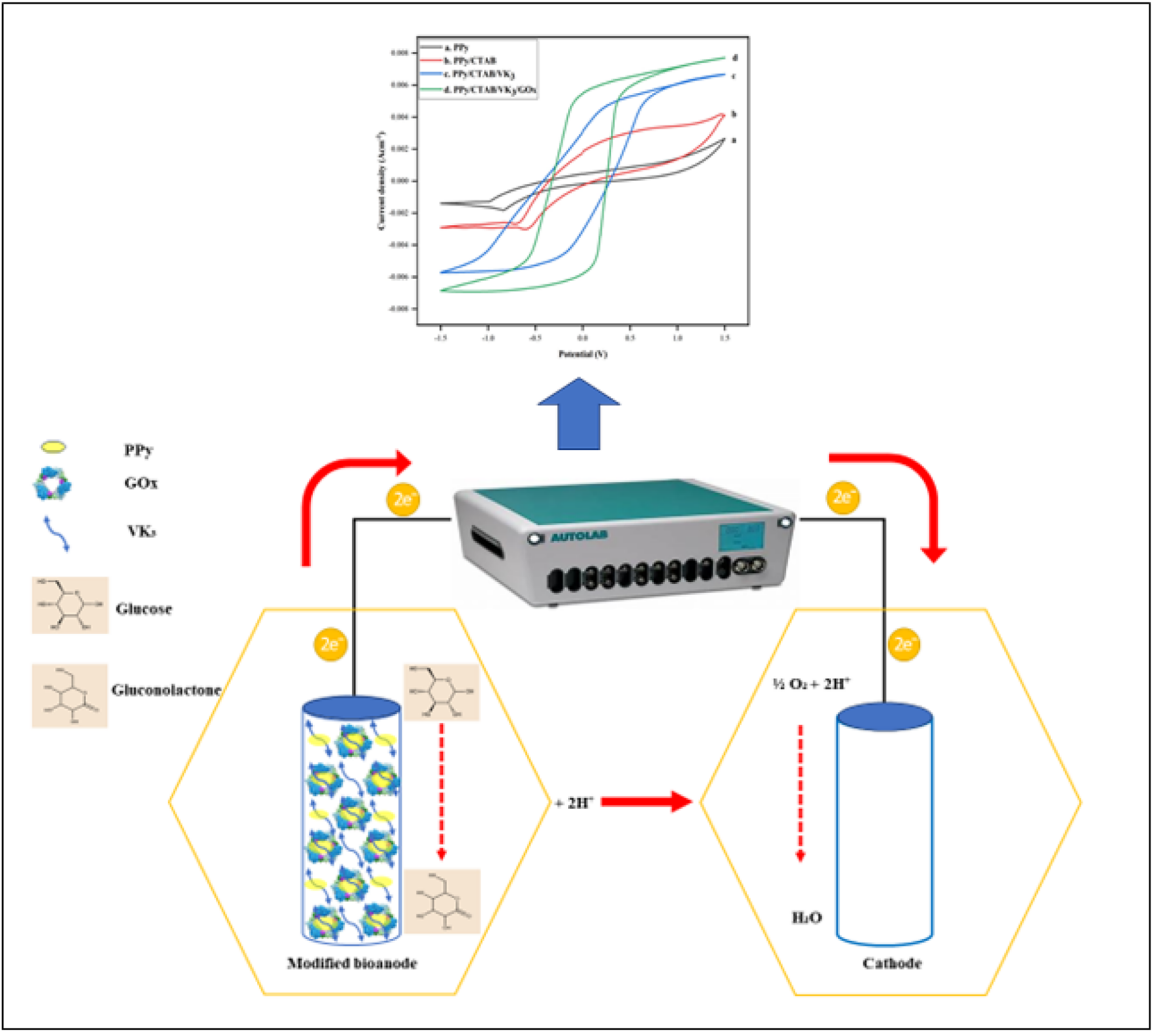
Schematic illustration of enzymatic biofuel cells (EBFCs).

Unprecedented population expansion has given rise to problems associated with the survival of mankind including increased pollution levels resulting in global warming; and a sharp fall in the non-renewable energy resources on mother Earth. These issues highlight the need to create alternate pathways in the realm of renewable energy technology. Fuel cells are primary electrochemical cells that efficiently transform the chemical energy of fuels into electrical power^1^ and have gained appreciable attention in the past few decades supporting their transition to a sustainable energy system^2^. Fuel cells may be broadly categorized into two viz. high-temperature and low-temperature fuel cells. The former kind includes solid oxide fuel cells (SOFCs), molten carbonate fuel cells (MCFCs), and proton exchange membrane fuel cells (PEMFCs); which operate at temperatures above 100 °C. The latter, biofuel cells (BFCs) in general; microbial biofuel cells (MBFCs) and enzymatic biofuel cells (EBFCs) in particular, are bioelectronic devices working in accordance with the physiological pH and the human body temperature, making them suitable candidates to supply power in biomedical devices^3^, consumer electronics, environmental monitoring, and biohydrogen production^4^. EBFCs show better compatibility with the human body; unlike MBFCs which may pose health risks associated with microbes^5^. EBFCs are considered reliable and economical for power production in implantable and wearable devices such as pacemakers, metabolite biosensors, pumps, etc. They employ reusable biocatalysts and renewable sources of energy such as glucose, fructose, methanol and ethanol while offering advantages such as low operating potentials^6^ and substrate specificity of catalysts which in turn helps to avoid the use of separators in between the electrodes in the electrochemical cell and conceptually helps in miniaturization of fuel cells^7^. A schematic illustration of EBFCs is presented in Fig. 1.

Extensive research is being carried out to explore the electrical connections of the redox enzyme biocatalysts with the bioelectrode surface^8^. Electrical communication is generally provided by either immobilizing the redox enzymes in electroactive polymers^9^ or by confining the electroactive relay to redox proteins^10^. Enzyme immobilization is one of the most crucial architectures of the bioelectrodes, particularly governing the biofuel cell performance. Immobilization of enzymes is performed to ensure continuous availability of enzymes for their frequent use and to avoid washing them off. If enzyme immobilization is rendered inefficient, considerable electron transfer becomes difficult to establish between the electrode surface and the active site of the enzymes. Simonas et al.^11^ employed semiconducting materials, titanium dioxides to enhance the biocatalytic performance of the biosensors. But in this scenario, conducting polymers are the most suitable materials to extend the analytical characteristics of biofuel cells and sensors^12^. Conducting polymers exhibit good electrical conductivity and exceptional capabilities to transfer electrons from enzyme-active sites towards electrodes. Conducting polymers like polyaniline, polypyrrole, polythiophene, etc., can successfully be employed in the charge transfer between the redox active center of the enzyme and the electrode^13^. The development of a bioanode with electropolymerized PPy adsorbed on the electrode surface is reported in this study. Herein, PPy plays a dual role as the charge transfer chain and in immobilizing GOx within its matrix. PPy acts as a conductive filler improving the transfer of electrons between the electrode and the redox-active center of GOx. Conductive PPy has been chosen in the design of this bioanode because of its remarkable qualities, including excellent thermal stability^14^, modifiable electrical conductivity, good environmental stability^15^, significant redox activity, biocompatibility^16^, ease of chemical and electrochemical preparation in a variety of organic solvents and aqueous solutions. This study also disclosed a straightforward and easy method for altering the size and morphology of PPy by involving a cationic surfactant, CTAB, acting as a structure-directing agent^17^.

In general, two major processes are employed in the transfer of electrons i.e. direct electron transfer (DET) and mediated electron transfer (MET)^18^. In the former kind, electron transfer occurs directly from the active site of the enzyme to the electrode without the utilization of a mediator^19^. The effectiveness of this transfer is greatly influenced by the orientation of the enzyme; to maintain a minimum electron tunneling distance, the enzyme must be immobilized within a matrix near the electrode. In this study, PPy embedded on the novel metal platinum (Pt) enhances DET because of the high surface area, modest biocompatibility, environmental and chemical stability of Pt. Platinum is largely employed in the fabrication of sensing systems due to its swift electron communication features, providing a desirable environment for charge transfer to occur between the active sites of the enzyme and the electrode^15^. It should be noted that the redox-active sites of the majority of enzymes are firmly embedded and concealed within their proteinaceous pockets. This means that even if these redox proteins are immobilised near the electrode surface within the CP-based matrix, electron transport from them is unlikely.

This critical problem of charge transfer between immobilised GOx and the electrode surface can be solved by the application of soluble redox mediators^13^. The employment of shuttle-like redox mediators can circumvent DET's shortcomings caused by long electron tunneling lengths that prevent the establishment of a strong electrical link between the enzyme and the electrode surface. These redox-active materials like methyl viologen^20^, ferrocene, ferritin^21^, etc. facilitate considerable charge transfer between the active site and electrode through a minimum distance using an electron hopping mechanism^22^. Mediators of low molecular weight and small size that require low over potential are preferred to allow swift electron transfers between the active site of enzyme and electrode surfaces with minimum power losses^23^.

Sema et al.^24^ designed a bioanode (manganese (IV) oxide nanoparticle /GOx/ glassy carbon paste electrode), wherein p-benzoquinone was utilized as a mediator for the transfer of electrons between GOx redox center and the electrode using glucose as the substrate. But the use of such non-biocompatible, non-biodegradable, and toxic mediators to tunnel electrons^25^, limits the use of these anodes in implantable and wearable devices. In this study, vitamin K_3_ offers a plausible solution in the construction of a biocompatible, and implantable fuel cell due to its environmentally inert, non-toxic, eco-friendly properties^26^. As a result, the inclusion of VK_3_ aids in the proper channeling of electrons between the deeply-seated redox active shells of GOx and the bioanode surface, resulting in enhanced electrochemical performance. VK_3_ also has a lower oxidation potential than other employed mediators like ferritin, thereby allowing it to be suitably used at the human body temperature. This proposed bioanode (PPy/CTAB/VK_3_/GOx) was fabricated to be used not only in the construction of EBFCs but also in the field of glucose detection technologies in biomedical applications. All the abbreviations and variables used in the paper are listed in Tables 1 and 2 respectively.

**Table 1:** List of abbreviations.

Abbreviation	Definition
BFCs	Biofuel cells
CE	Counter electrode
CTAB	Cetyltrimethylammonium bromide
CV	Cyclic voltammetry
DDW	Double distilled water
DET	Direct electron transfer
EBFC	Enzymatic biofuel cells
EIS	Electrochemical impedance spectroscopy
FTIR	Fourier transform infrared
GOx	Glucose oxidase
LSV	Linear sweep voltammetry
MBFCs	Microbial biofuel cells
MCFCs	Molten carbonate fuel cells
MET	Mediated electron transfer
PEMFCs	Proton exchange membrane fuel cells
PPy	Polypyrrole
Pt	Platinum
PTSA	*p*-toluenesulfonic acid
RE	Reference electrode
SEM	Scanning electron microscopy
SOFCs	Solid oxide fuel cells
TEM	Transmission electron microscopy
TGA	Thermogravimetric analysis
VK_3_	Vitamin K_3_
WE	Working electrode
XRD	X-ray diffraction

**Table 2:** List of variables used in the paper.

Variable	Definition	Unit
σ	Electrical conductivity	S/cm
G_7_(W/S)	Correction factor for non-conducting bottom surfaces	dimensionless
W	Thickness of the sample	cm
S	Spacing between the probes	cm
I	Current	A
V	Voltage	V
D	Crystallite mean size	nm
K	Shape factor	dimensionless
θ	Diffraction angle at maximum peak intensity	radian
λ	X-ray wavelength	nm
β	Full width at half maximum of the diffraction angle	radian

## Experimental section

Pyrrole (98%), (SRL, India) was distilled prior to use. Cetyltrimethylammonium bromide (CTAB) (98%) and *p*-toluenesulfonic acid (PTSA) (98%) were obtained from Sigma Aldrich, India. Vitamin K_3_ (98%), glucose oxidase (GOx) (activity 100,000–150,000 units g^–1^ of protein) from Aspergillus niger, phosphate buffer solution of pH 7.4, citrate buffer solution of pH 5, acetone (99%), ethanol (99.9%), sulphuric acid (97%), D-( +)-glucose anhydrous (99.5%), potassium ferrocyanide (98–102%), were used as received without further purification and were procured from Central Drug House, India. All of the reagents were of analytical grade. Double distilled water (DDW) was used throughout the experiment.

The electrochemical studies were performed using a conventional three-electrode assembly consisting of a platinum wire (0.5 mm diameter) as the working electrode (WE), KCl-saturated Ag/AgCl as a reference electrode (RE), and aluminium wire (0.91 mm diameter) as a counter electrode (CE). The three-electrode assembly was connected with a computer-controlled potentiostat/galvanostat (PGSTAT 302 N Autolab, Switzerland) for conducting the experiments. A four-in-line probe electrical conductivity-measuring instrument, SES Instruments, Roorkee, India, was used for measuring the DC electrical conductivity. The digital ultrasonic cleaner LT-350 (Labrotonics) was used for the cleaning of the electrodes. The morphological characterizations of the nanocomposite were carried out by scanning electron microscopy (SEM, Evo 18 Zeiss) and transmission electron microscopy (TEM, TECNAI G20 HR-TEM, Thermo Scientific) with samples positioned on a carbon-coated copper grid. The thermogravimetric analysis (TGA) and X-ray diffraction (XRD) were performed on DTG-60H (Shimadzu) and Rigaku (SmartLab), respectively. Fourier transform infrared (FTIR) spectroscopy was recorded by Nicolet iS50 FT-IR (Thermo Fisher Scientific) in the spectral range from 4000 to 400 cm^−1^. All the experiments were conducted at room temperature.

### Fabrication of the anode

**Figure 2:**
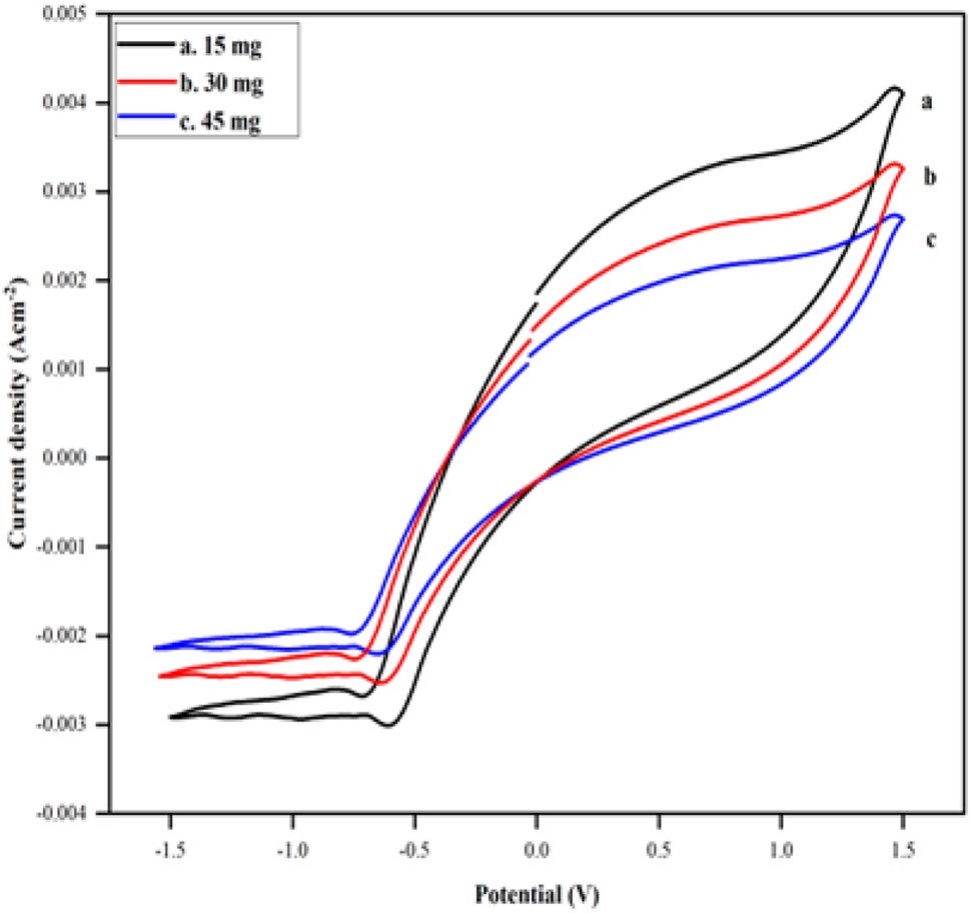
Cyclic voltammograms of PPy/CTAB in PBS of pH 7.4 at (a) 15, (b) 30 and (c) 45 mg of CTAB.

The platinum and aluminium electrodes were cleaned for 15 min in an ethanolic solution using the ultrasonic cleaner and were later rinsed with double-distilled water to remove the impurities. The electrodes were further polished by using the technique of cyclic voltammetry at a scan rate of 50 mV s^–1^ in 1.0 M sulfuric acid solution. The three-electrode electrochemical cell setup was then used to electrochemically polymerize pyrrole monomers on the working electrode. Pyrrole monomers (10 vol. % solution) were added in a drop-wise manner to a 0.1 M solution of *p*-toluene sulfonic acid (PTSA), acting as the dopant. The mixture was magnetically stirred for a period of 15 min until a homogenous solution was obtained. To optimize the concentration of CTAB to be used, different quantities of the surfactant (15 mg, 30 mg, 45 mg) were added to the pyrrolic solution and tested using cyclic voltammetry. As seen in Fig. 2, 15 mg of CTAB showed better results and was found optimum to be finally mixed thoroughly with the solution. This composition was left on the stirrer for 20 min until a grey-colored solution was obtained.

**Figure 3:**
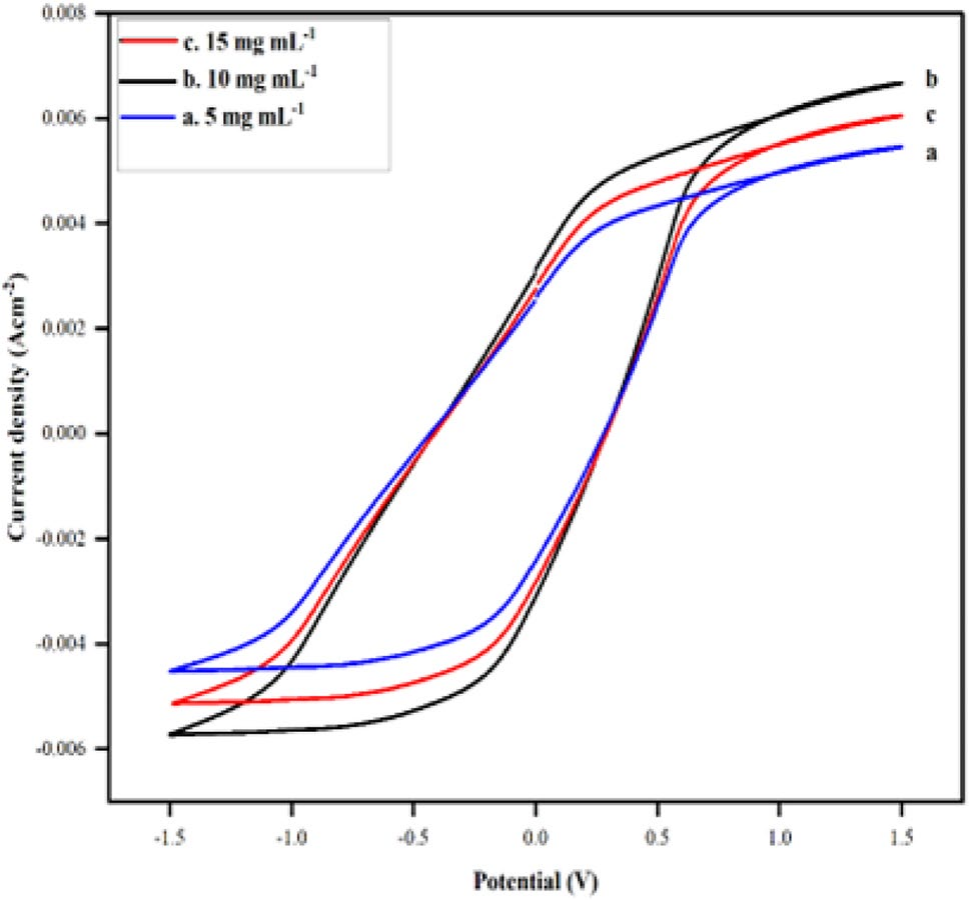
Cyclic voltammograms of PPy/CTAB/VK_3_ in PBS of pH 7.4 at (a) 5, (b) 10 and (c) 15 mg mL^–1^ of VK_3_.

**Figure 4:**
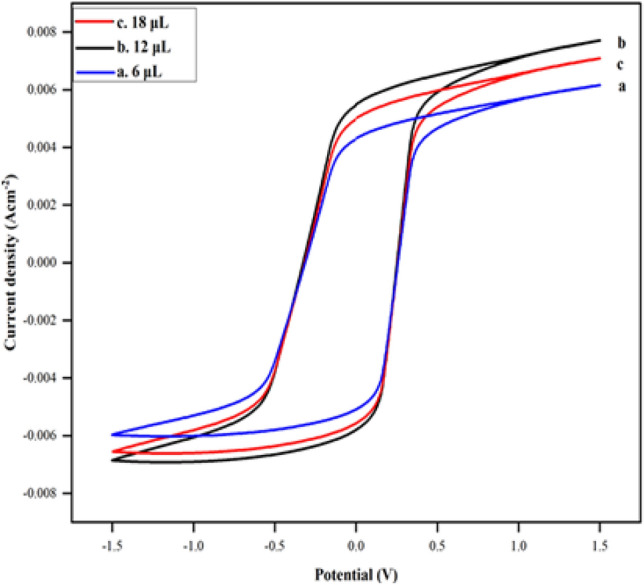
Cyclic voltammograms of PPy/CTAB/VK_3_/GOx in PBS of pH 7.4 at (a) 6, (b) 12 and (c) 18 μL of GOx.

For the bioanode construction, optimum concentrations of VK_3_ and GOx to be used were also determined using CV. The electrochemical cell was enriched with various quantities of GOx (10.0 mg mL^−1^ in a citrate buffer solution of pH 5.0) and VK_3_ for the entrapment of the same into the PPy moiety. Three different concentrations of VK_3_ (5 mg mL^−1^, 10 mg mL^−1^, 15 mg mL^−1^) and GOx (6 μL, 12 μL, 18 μL) were chosen for the optimization of this anode. It was observed from the CVs of Figs. 3 and 4, that the electrode having 10 mg mL^–1^ of VK_3_ and 12 μL of GOx showed better electrochemical performance. After the addition of the mediator and enzyme to the mixture, the cell was placed in the potentiostatic set-up and electrochemical polymerization was carried out in the potential window of − 1.5 to 1.5 V using cyclic voltammetry. The fabricated electrode after polymerization was immediately rinsed with acetone, ethanol, and deionized water, respectively, to remove weakly adsorbed polymer and residual monomers, if any, and later left to dry for six hours in the oven at 35 °C.

### Electrical conducting behaviour of polypyrrole

Electron transport can be classified into the following types: in metallic conductors, the electrical conductivity (σ) increases linearly with decreasing temperature, and in semiconductors, conductivity increases generally with an increase in temperature^27^. The electrochemically synthesized PPy was scraped off from the electrode and placed in the oven for overnight drying at a temperature of 40 °C. After drying, 250 mg of the sample was finely ground into fine powder in a mortar and pestle. This powder was then placed into a pellet-making die set in a hydraulic press at 50 KN for 20 min. A micrometer/screw gauge was then used to measure the thickness of the prepared sample. The conductivity of the pressed pellet sample was measured by using a four-in-line-probe technique of conductivity measurement for semiconductors. The four-probe assembly consists of a probe station (four probe tips), a constant current source, a PID-controlled oven, and a microvoltmeter. Two of the four probes are used to source current, and the other two are used to measure voltage, which means as current flows between the outer probes, the voltage drop across the inner probe is determined. The various tests performed on the PPy pellet were isothermal, cyclic, and stability tests. The sample under observation was placed on a disc of the four-probe arrangement with the probes resting precisely in the sample's center. The probes were tightened to produce a very gentle pressure on the sample without piercing it. This setup was then placed in the oven of the four-probe system, with the current passing through the outer probes, the floating potential across the inner pair of probes was recorded. To increase the temperature of the system, the oven was switched on, and the resulting current and voltage were evaluated.

## Results and discussion

The four-in-line-probe method was utilized to determine the electrical conductivities of the synthesized PPy sample. Due to its high accuracy and easy sample preparation, this four-probe method is the most common technique to measure a semiconductor material’s conductivity^28^. To minimize the contribution of protonic conductivity to the total electrical conductivity, the samples were thoroughly dried before pellet-making to ensure ideal results. The conductivity measurements were done under ambient conditions and the following equation was used to calculate the electrical conductivity of the sample:

1 $$\sigma =\frac{\sigma 0 }{{G}_{7 }(\frac{W}{S}) },$$

where σ is the electrical conductivity (S/cm), G_7_(W/S) is the correction factor for non-conducting bottom surfaces, which is a function of W, the thickness of the sample under observation (cm), and S is spacing between the probes (cm);

2 $${G}_{7}\times \left(\frac{W}{S}\right)=\frac{2S\left({\text{ln}}2\right)}{W},$$

and

3 $${\sigma }_{0}=\frac{I}{(V \times 2\pi S)},$$

where I is the current (A) and V is the voltage (V).

**Figure 5:**
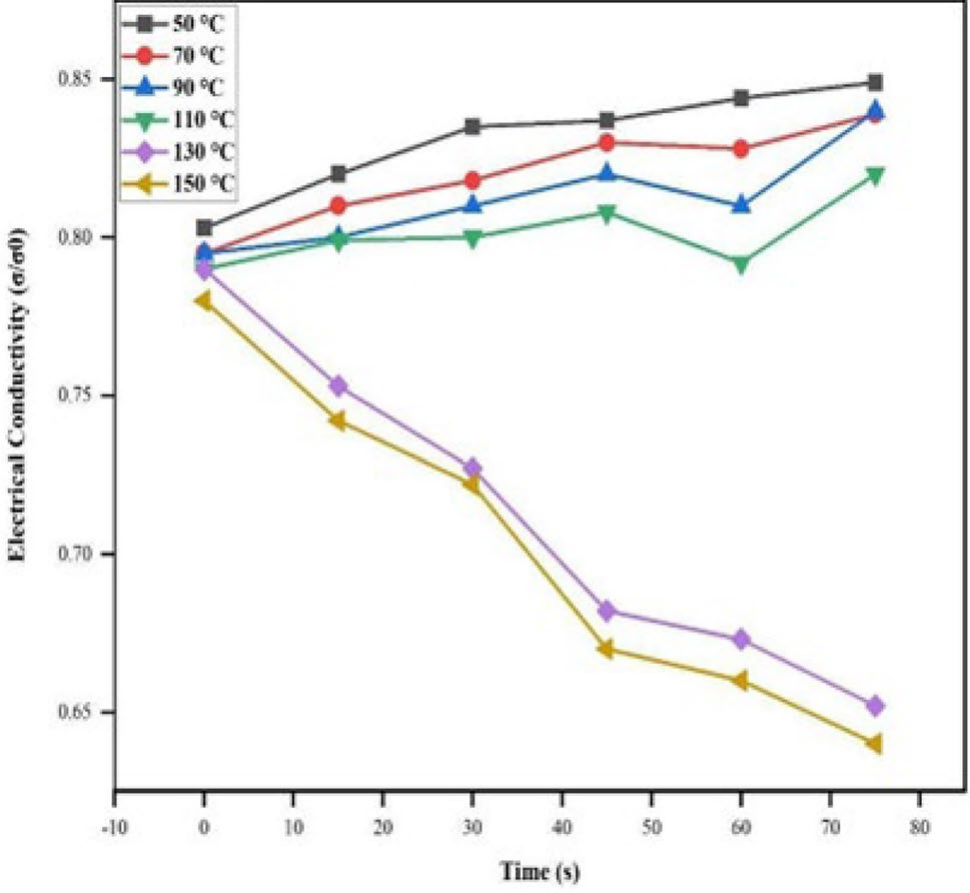
Isothermal stability of PPy in terms of retention of dc electrical conductivity with time at 50, 70, 90, 110, 130, and 150 °C.

**Figure 6:**
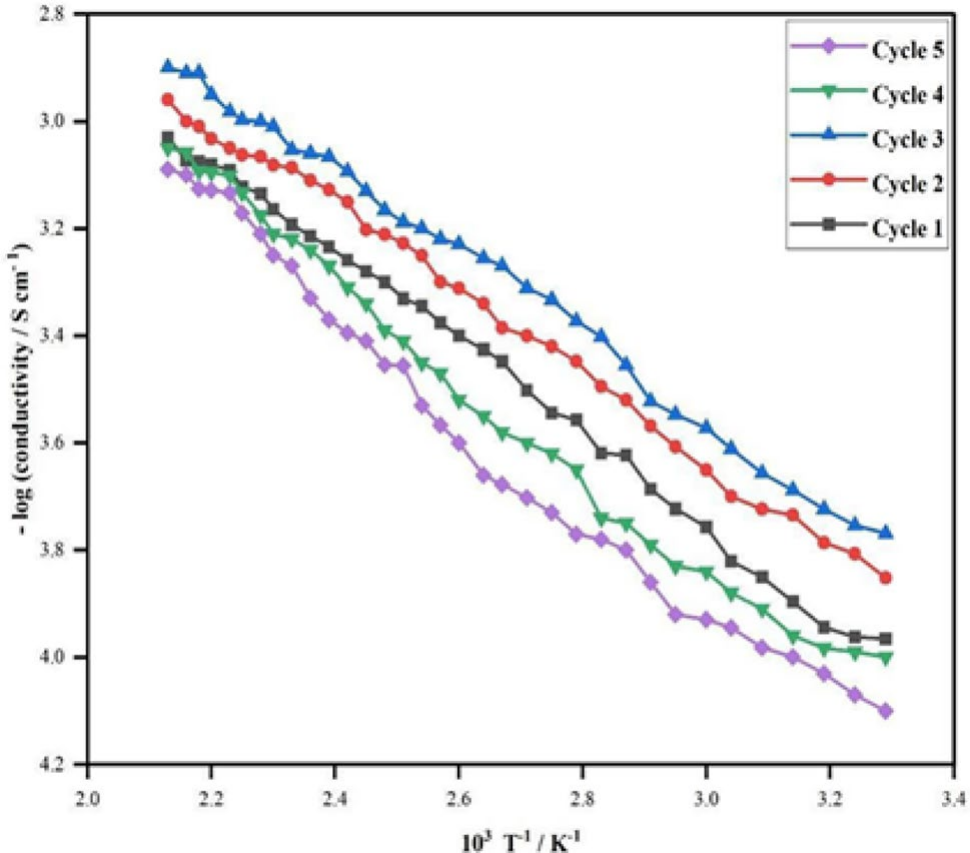
Arrhenius plot of retention of dc electrical conductivity for PPy during heating–cooling cycles up to 200 °C.

**Figure 7:**
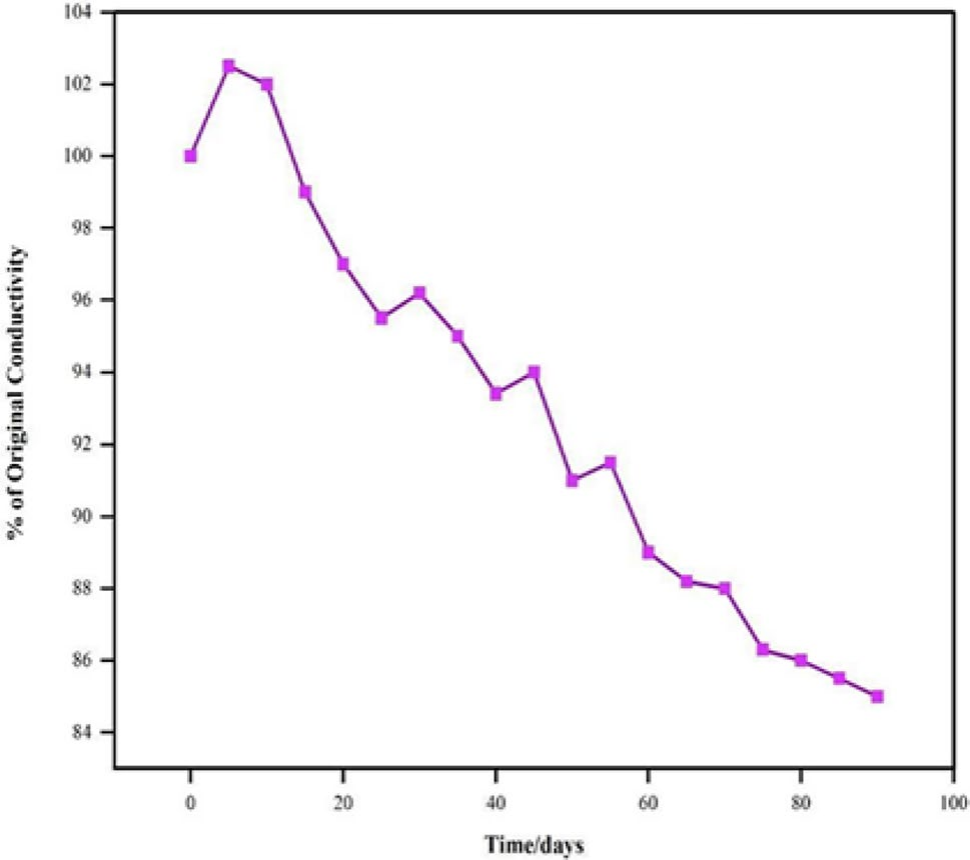
Conductivity versus time of exposure to laboratory air for PPy material.

The variation of electrical conductivity (σ) of the prepared sample was investigated at temperatures over a range of 35–200 °C. Under isothermal conditions at temperatures 50, 70, 90, 110, 130, and 150 °C, the thermal stability of the material in terms of DC electrical conductivity retention was studied at intervals of 15 min. The results are shown in Fig. 5. To interpret the graph, the electrical conductivity is quite stable at temperatures 50, 70, 90, and 110 °C proving that the conductivity of the sample is significantly stable under ambient temperature conditions. At 130 and 150 °C, the decrease in conductivity seen may be due to the loss of the dopant and a reaction of the dopant with the material. To monitor the stability of the material (HCl treated) in terms of electrical conductivity retention, five heating cycles (up to 200 °C) were recorded by repeated linear four-in-line-probe DC electrical conductivity measurements for increasing temperatures at 1 h intervals. The electrical conductivity for each cycle was plotted as log σ versus 1000/T (T in K) as shown in Fig. 6. The temperature dependence of each plot can be seen to follow the Arrhenius equation with only minor differences in five heating cycles, this proves the optimum stability of the sample during the heating–cooling cycles under extreme oxidizing conditions. Figure 7 shows that the room temperature conductivity is minimally affected by short-term exposure to laboratory air, making PPy a stable material. These results prove that the electrically semiconducting behavior of PPy can be useful in bioelectronic devices such as EBFCs to provide electrical communication to the shuttling electrons passing through PPy to the electrode surface.

**Figure 8:**
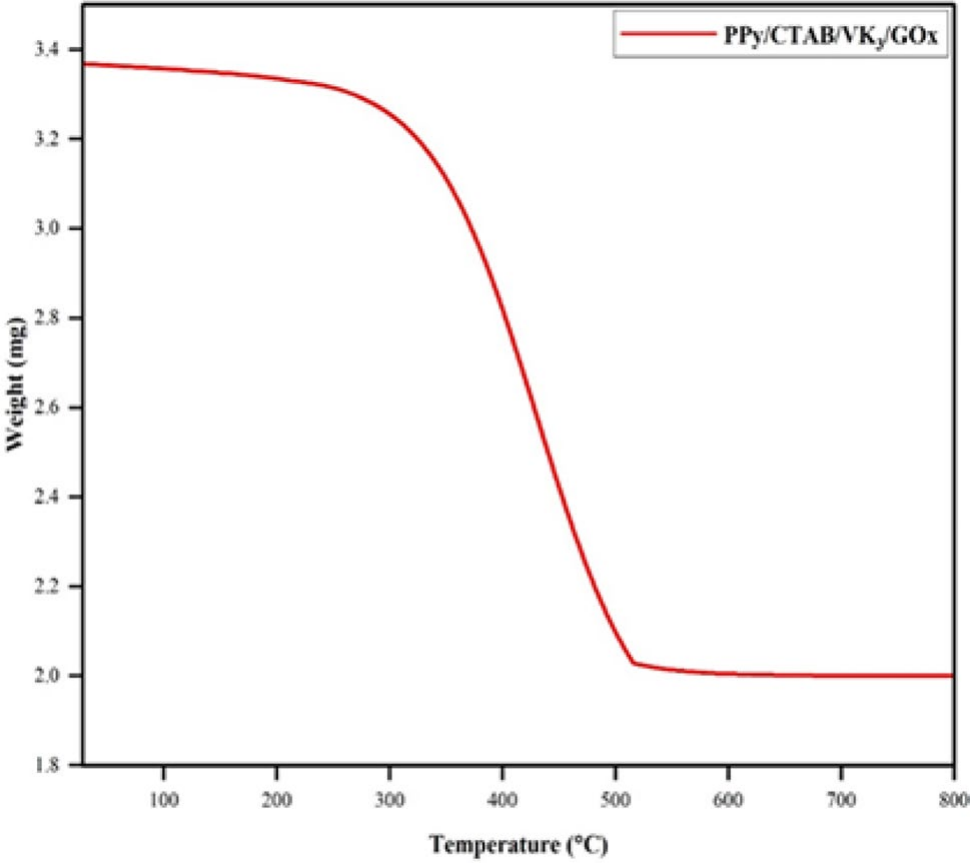
TGA curve of PPy/CTAB/VK_3_/GOx.

Thermal stability is one of the most important characteristics to be noted of polymeric materials. The TGA of the biocomposite PPy/CTAB/VK_3_/GOx is shown in Fig. 8. The initial weight loss (80–100 °C) results due to the evaporation of water molecules. Subsequent weight loss to around 300 °C could be credited to the evaporation of other volatile components. The thermogram shows stability up to 300 °C after which a steep decomposition takes place. This weight loss (from 300 to 520 °C) can be attributed to the degradation of PPy backbone. The decomposition terminates at around 600 °C leaving behind a carbon-black mass.

**Figure 9:**
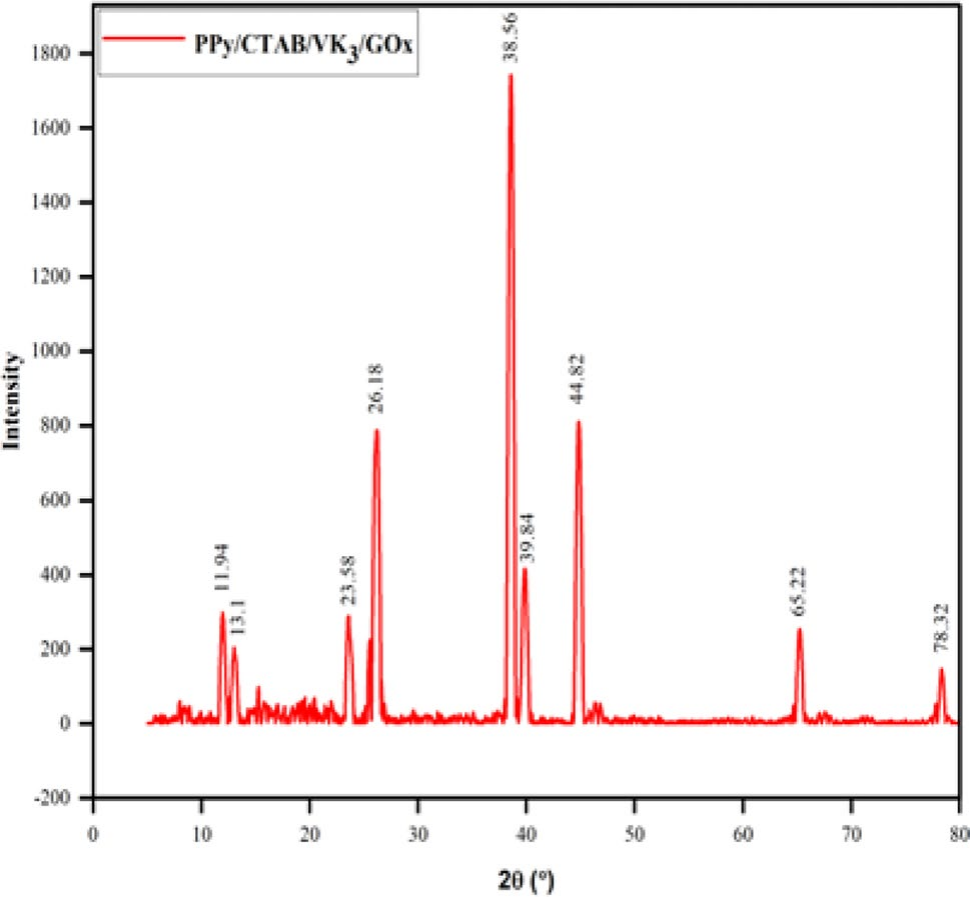
XRD of PPy/CTAB/VK_3_/GOx.

XRD analysis was carried out at a scan rate of 10 degrees/min. As evident in Fig. 9, the PPy/CTAB/VK_3_/GOx bioanode displays sharp peaks at 2θ suggesting the crystalline nature of the prepared biocomposite. The polymer chains are distinctly oriented due to the repeating units of pyrrole rings. This shows that the presence of CTAB imparts crystallinity (at 2θ = 26.16°) to the often-amorphous character of PPy^29^. VK_3_ exhibits several peaks in the 2θ region of 15–30° which is in accordance with the established XRD pattern obtained for VK_3_^30^. The crystallinity of GOx is established from the peak located at 2θ = 22.5°^31^.

Scherrer’s formula Eq. (4) is used to calculate the average crystal size of PPy from the evident sharp peaks from the XRD pattern.

4 $$D=\frac{K\lambda }{\beta cos\theta },$$

where D is the crystallite mean size (nm), K is the shape factor (dimensionless and has a value of 0.89 for unknown shape), θ is the diffraction angle at maximum peak intensity, λ is the X-ray wavelength and β is the full width at half maximum of the diffraction angle (in radians). The calculated crystal size comes out to be 30.12 nm.

**Figure 10:**
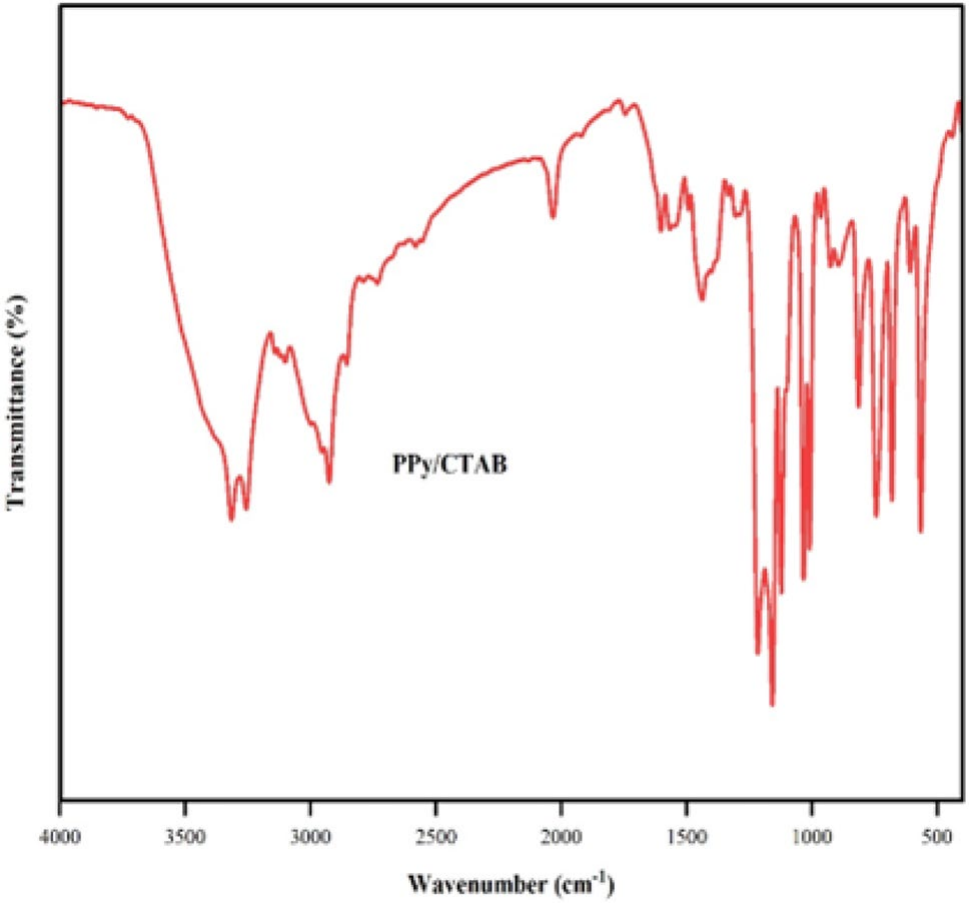
FTIR spectrum of PPy/CTAB.

The FTIR spectrum of the PPy-CTAB biocomposite is given Fig. 10. The absorption peak around 3400 cm^–1^ originates from the stretching vibrations along the O–H axis, indicating the presence of moisture in the PPy/CTAB composite. The peaks at 1600 and 3320 cm^–1^ are linked to the fundamental stretching vibrations of C=C and N–H in the pyrrole ring. The distinctive peak at 1315 cm^–1^ corresponds to the C–N bond within the ring. The in-plane deformation of C–H is reflected in the maxima at 1200 cm^–1^. Additionally, the peaks at 811 and 920 cm^–1^ signify the C–H wagging of the ring^32^. The absorption signals at 2920 and 2853 cm^–1^ confirm the presence of CTAB, corresponding to the asymmetric and symmetric stretching modes of methyl and methylene groups in CTAB. Thus, the FTIR results validate the successful synthesis of the PPy-CTAB biocomposite.

**Figure 11:**
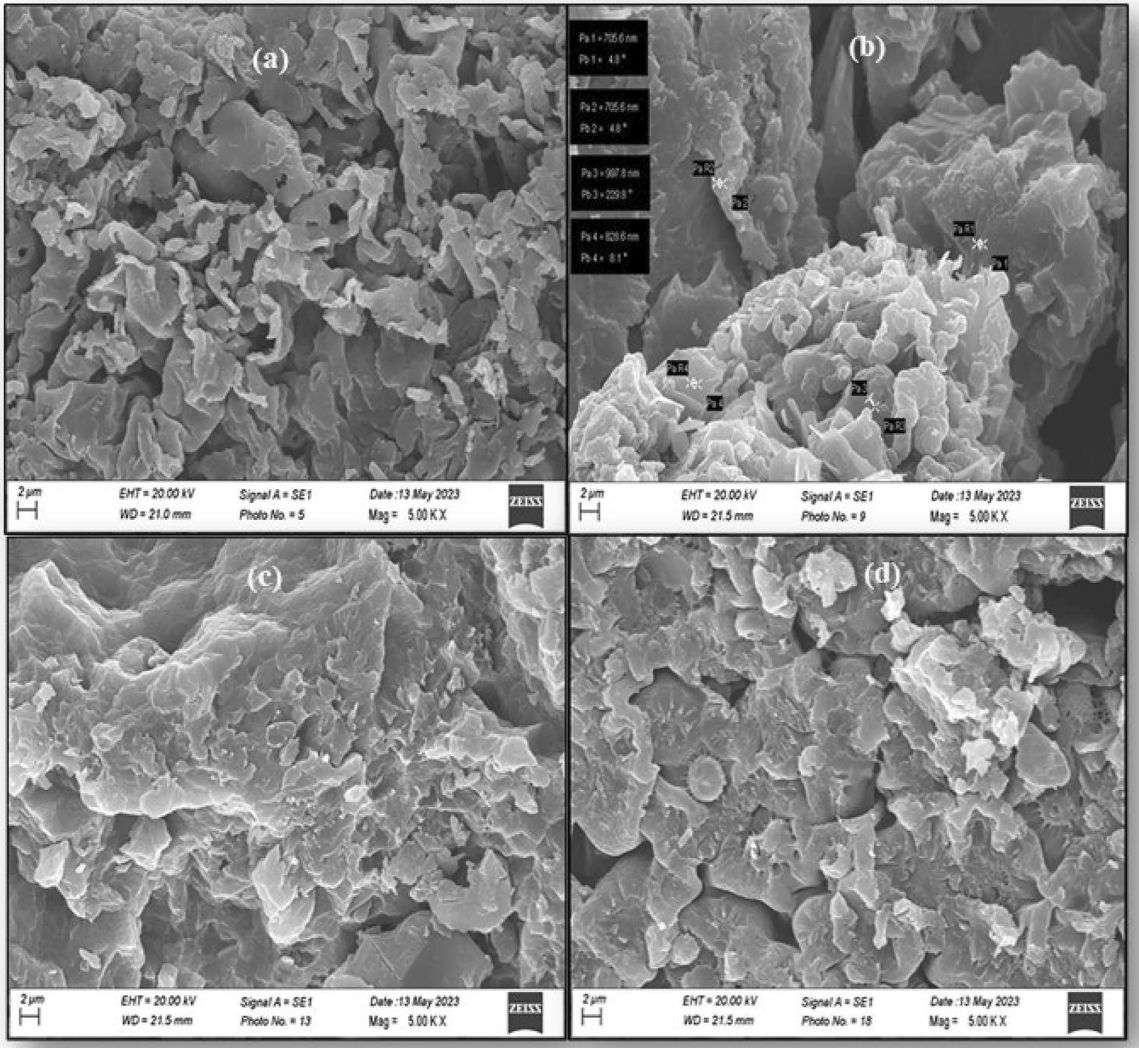
SEM micrographs of (**a**) PPy, (**b**) PPy/CTAB, (**c**) PP/CTAB/VK_3_, (**d**) PPy/CTAB/VK_3_/GOx.

**Figure 12:**
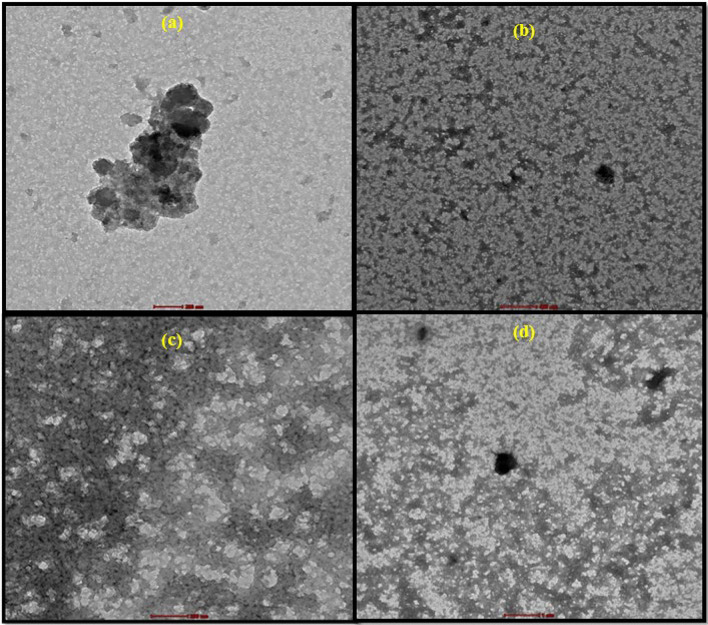
TEM micrographs of (**a**) PPy, (**b**) PPy/CTAB, (**c**) PPy/CTAB/VK_3_, (**d**) PPy/CTAB/VK_3_/GOx.

SEM and TEM were carried out to examine the morphologies of PPy, PPy/CTAB, PPy/CTAB/VK_3_, and PPy/CTAB/VK_3_/GOx biocomposites as displayed in Figs. 11 and 12. Figure 11a showed flake-like structures indicating the presence of PPy. It was observed from Fig. 11b that the fibrous structure of PPy is altered into a granular bead-like form with the beads showing agglomeration on the addition of the surfactant, CTAB, while Fig. 11c and d displayed the superficial characteristics of the bioanode and manifested notable differences proving the addition of VK_3_ and GOx to the biocomposite, resulting in the construction of a dense network on the surface. On comparing, Fig. 12a and b, it is evident that on the addition of CTAB, a rough matrix has been developed. TEM results helped to validate an intimate association among the components of the biocomposite.

**Figure 13:**
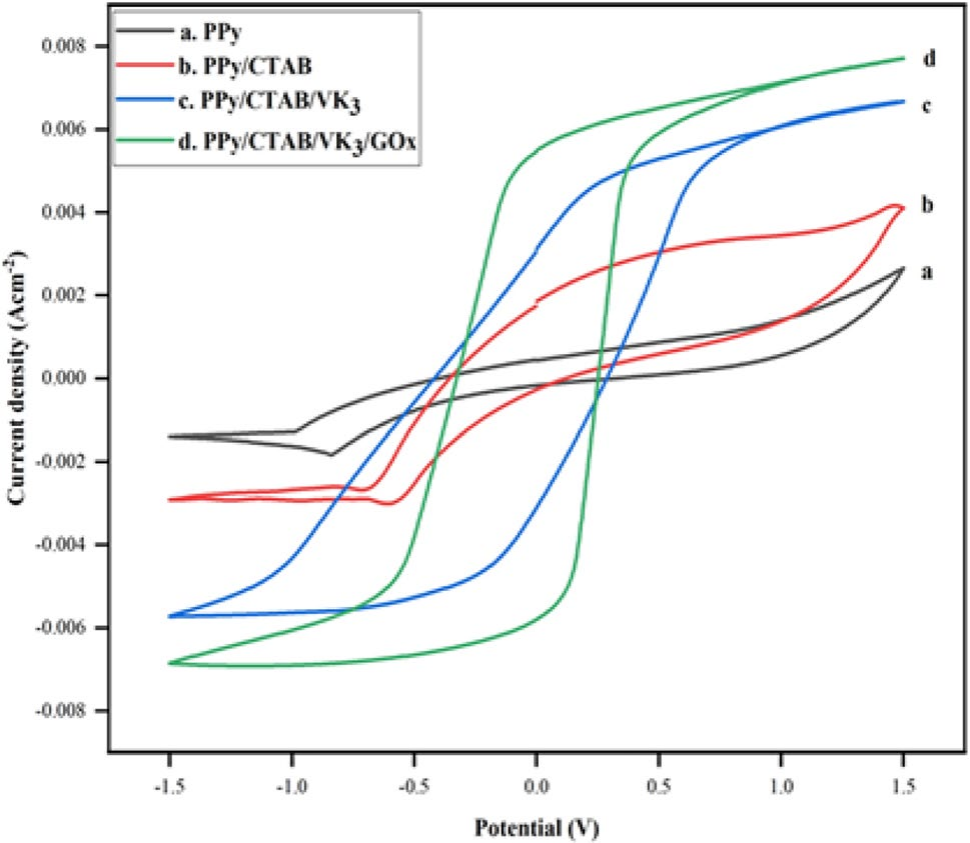
Cyclic voltammograms of PPy, PPy/CTAB, PPy/CTAB/VK_3_, PPy/CTAB/VK_3_/GOx in PBS of pH 7.4 at a scan rate of 100 mV s^–1^.

The prepared biocomposite provided electrical communication between the deeply-seated active sites of the enzyme and the electrode surface by utilizing the polymer PPy and simultaneously enhancing the shuttling of electrons through the mediator, vitamin K_3_. Controlled experiments were performed to observe and study this electrical communication. The cyclic voltammograms in Fig. 13, were recorded over a potential window of − 1.5 to 1.5 V at a potential scan rate of 100 mV s^–1^ in N_2_-purged PBS of pH 7.4. Very low current density and absence of any remarkable peak were observed in curve (a), but curve (b) showed comparatively higher current density owing to an increase in the surface area on the addition of CTAB, resulting in increased catalytic activity. The anodic current density of (c) on the addition of VK_3_, jumped to a reading of 4.13 from 3.03 mA cm^–2^, proving that the VK_3_ moiety dramatically enhances the redox current of the bioelectrode due to the fast electron transfer within the electrolyte electrode interface. These observations are consistent with the results of Ruma et al.^25^ wherein the integration of a biocompatible redox mediator Ferritin into the PPy matrix enhanced the overall current density of the prepared anode. It was observed that the bioelectrode composition of PPy/CTAB/VK_3_/GOx displayed the best catalytic activity with the highest anodic current density of 5.09 mA cm^–2^ effectively and reproducibly. This advancement can be attributed to the electrical communication and efficacious electron transport occurring between the deeply embedded redox cofactor of GOx and PPy/CTAB which was mediated through VK_3_.

**Figure 14:**
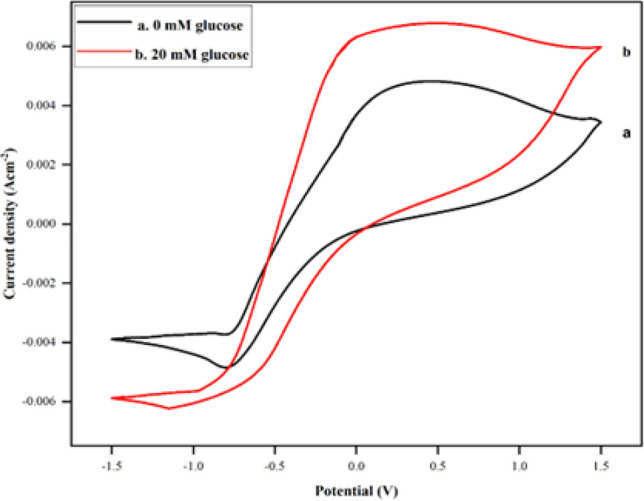
CVs of PPy/CTAB/VK_3_/GOx in PBS of pH 7.4, (a) in 0 mM and (b) 20 mM glucose at a scan rate of 100 mV s^–1^.

The bio electrocatalytic activity of the bioanode PPy/CTAB/VK_3_/GOx in PBS towards glucose oxidation was investigated in the presence (20 mM) and absence of glucose in the potential window of − 1.5 to 1.5 V as shown in Fig. 14. It was found that in the presence of glucose, the fabricated bioanode produced a higher anodic current density implying that it shows considerable catalytic activity for glucose oxidation. This result is in line with the observations of Won-Yong et al.^33^ where the glucose dehydrogenase (GDH) modified anode showed an increased catalytic current in the presence of glucose, suggesting that the anode utilizes glucose to generate current in a GDH-enzyme amount-dependent fashion. The CV showed a magnified current at 6.35 mA cm^–2^ with apparent reversible oxidation–reduction peaks. These peaks promote the idea of an easy and efficient electronic transition (due to the presence of the pendant VK_3_ moiety) between the concealed redox-active cofactor of GOx and the carrier electrode. Also, it can be noted that in the absence of glucose, the oxidation peak appeared at around 0.49 V, corresponding to the reduction peak at − 0.55 V, contrarily in 20 mM glucose, the bio composite oxidizes glucose at a comparatively lower oxidation potential of about 0.37 V and the reduction potential of − 0.5 V. In the vicinity of glucose, GOx present at the modified electrode surface catalyzes the oxidation of glucose thereby lowering the oxidation potential. The electrocatalytic performance of PPy/CTAB/VK_3_/GOx is compared with previously published work in Table 3.

**Table 3:** Comparison of PPy/CTAB/VK_3_/GOx with similar bioanodes.

Bioanode	Current density (mA cm^−2^)	References
GCE/PPy/Frt/Di–NADH–GDH	1.2	^35^
CS/n(GOx-PPy)/n(GOx-PPy)/cMWCNTs/GCE	0.691	^37^
GCE/3DG-GOx	0.07	^38^
GCE/PNT-CHI/FDH	2.45 ± 0.39	^39^
GCE/PPy/Au/CNT@Fe_3_O_4_/Frt/GOD	6.01	^25^
GC-Ppy-Ag-GO/Frt/GOx	5.7	^40^
KB/PLL-VK_3_/Dp/GDH	2	^26^
PAA/VK_3_–Dp/GDH	0.65	^41^
PPy/ CTAB/VK_3_/GOx	6.35	This work

*GCE* glassy carbon electrode, *Frt* ferritin, *NADH* nicotinamide adenine dinucleotide, *GDH* glucose dehydrogenase, *MWCNTs* multi-walled carbon nanotubes, *3DG* three-dimensional graphene, *PNT* polypyrrole nanotubes, *CHI* chitosan, *FDH* fructose dehydrogenase, *Au* gold nanoparticles, *CNT* carbon nanotubes, *Fe*_3_*O*_4_ iron oxide, *Ag* silver nanoparticles, *GO* graphene oxide, *KB* Ketjenblack, *PLL* poly-l-lysine, *Dp* diaphorase, *PAA* polyallylamine.

**Figure 15:**
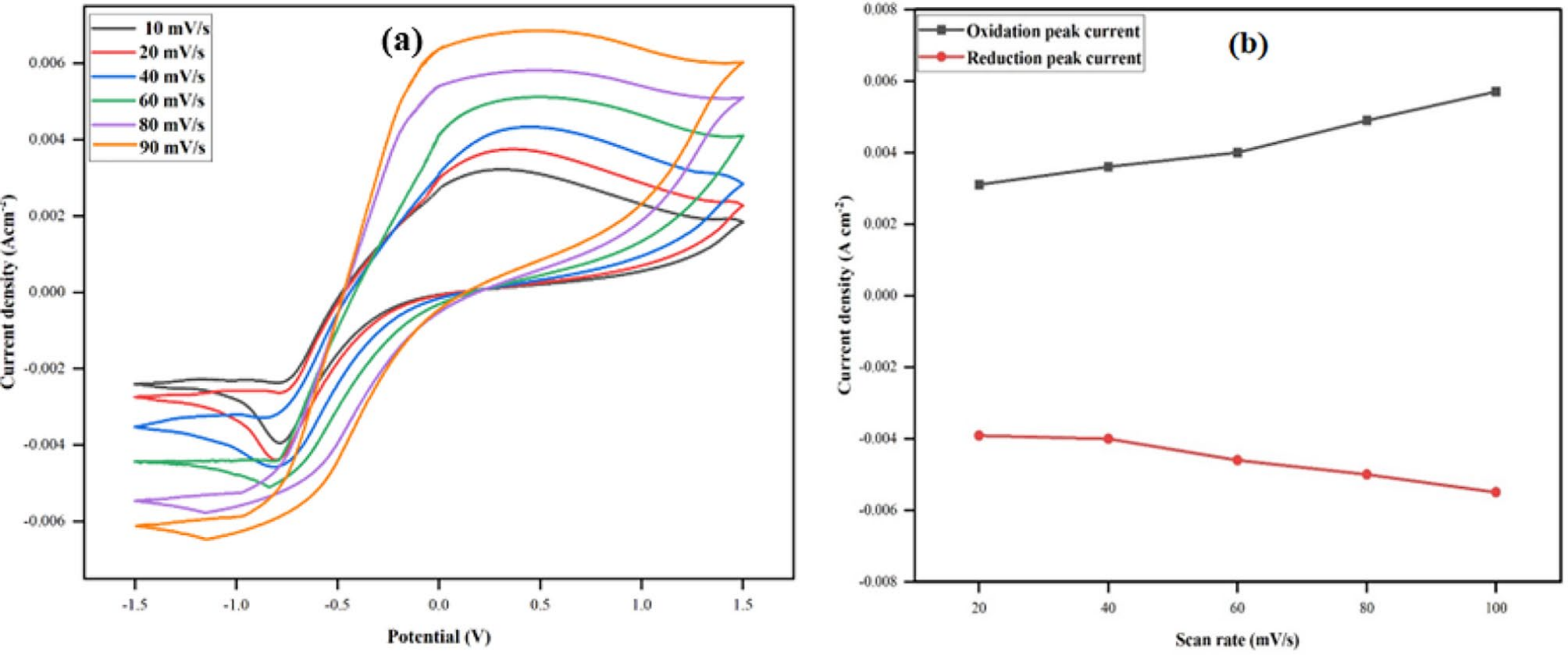
(**a**) CVs of PPy/CTAB/VK_3_/GOx in PBS of pH 7.4 at scan rate from 10 to 100 mV s^–1^ (from inner to outer); (**b**) plot of peak current vs. scan rate.

The cyclic voltammograms in Fig. 15a provide an account of the effect of scan rates from 10 to 100 mV s^–1^ over the potential window of − 1.5 to 1.5 V, on the electrocatalytic performance of the bioanode. The results reveal that the fabricated bio anode pertains to satisfactory electrochemical performance in the wide range of said scan rates. A proportional relationship can also be drawn out between the electrocatalytic current and scan rate. A similar relationship between the scan rate and the produced current output of the constructed anode (chitosan (CHI)@reduced graphene (rGO)-polyaniline (PAni)/ferritin (Frt)/GOx) was reported by Sufia et al.^34^. The quick hike in the concentration gradient within a short period leads to a swift response of the electrode to the electrocatalytic processes thereby showing a straight-line correspondence of the peak current with the scan rate and suggesting a diffusion-controlled procedure. These results also suggest the perfect immobilization of GOx on the electrode and the ability of the porous electrode material (due to increased surface area), to furnish a high electrode–electrolyte net contact area accompanying the reduced charge diffusion pathway for electrolyte ion conduction. The corresponding calibration curve, Fig. 15b, shows a linear relationship between the respective current densities and increasing scan rates, demonstrating the manufactured anode’s swift catalytic reaction. The linear regression equations: I_pa_ (oxidation peak current) = 0.0007x + 0.0023, I_pc_ (reduction peak current) = − 0.0004x–0.0033, and the correlation coefficient (R^2^) values of 0.9753 and 0.9674 of the redox couples are also calculated.

**Figure 16:**
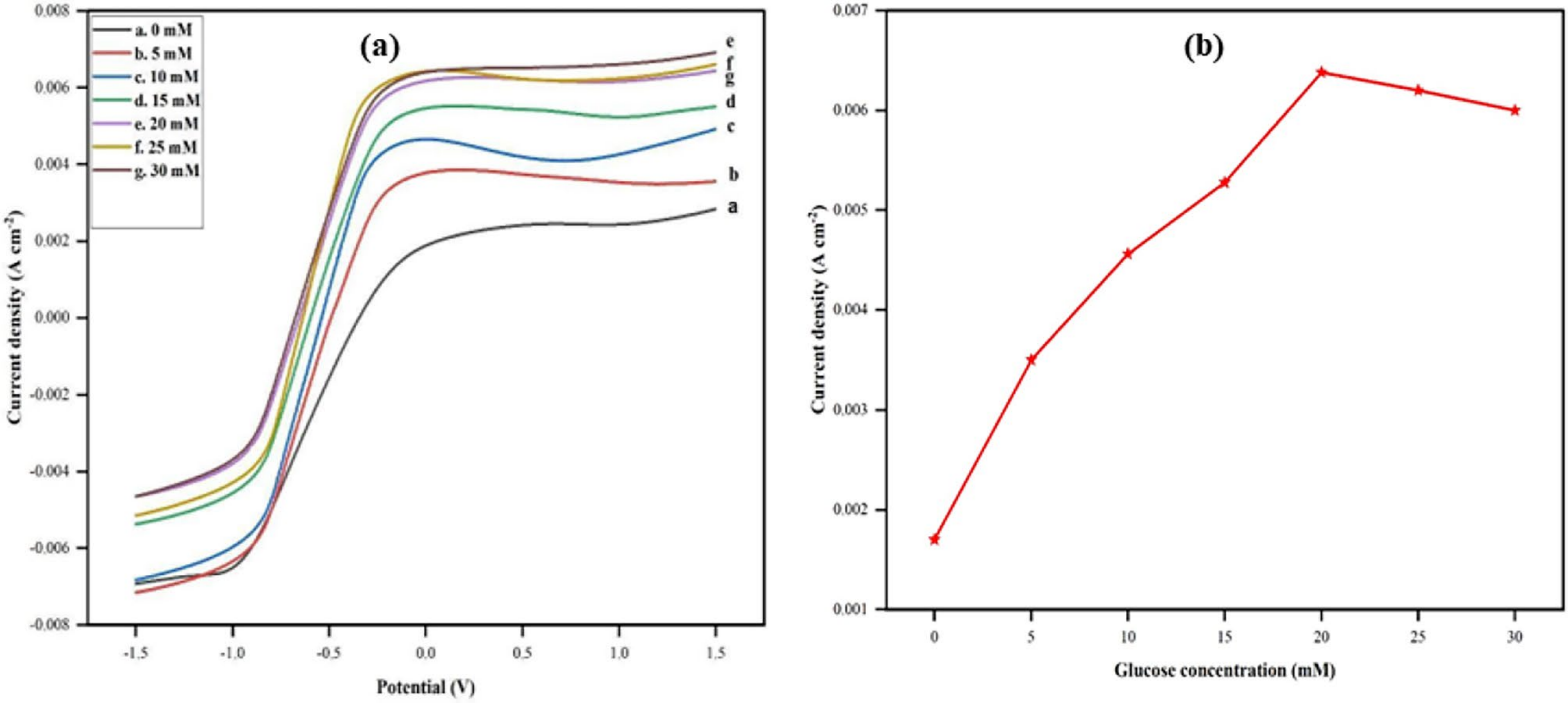
(**a**) Linear Sweep Voltammograms of PPy/CTAB/VK_3_/GOx in PBS of pH 7.4 at different concentrations of glucose; (**b**) the calibration curve corresponding to the electrocatalytic current against the variable concentration of glucose.

To study the bioelectrocatalytic oxidation currents generated by the PPy/CTAB/VK_3_/GOx fabricated bioanode concerning varying concentrations of glucose (0 to 30 mM) in PBS, an LSV investigation was performed. As is evident from Fig. 16a, the current densities increased linearly with a gradual increase in the concentration of glucose up to 20 mM, reaching a saturation limit of 6.35 mA cm^–2^. This increase in current can be attributed to the appreciable electrocatalytic performance of the bioanode enhanced by the presence of PPy providing extensive electrical communication and the mediator VK_3_ which aided in the easier shuttling of electrons from the enzyme active site to the modified electrode. Also, such peaks are indicative of sufficient loading of the immobilized GOx enzyme on the electrode. It can also be observed that the current density beyond this saturation limit decreases gradually owing to the hindrance caused by higher glucose concentrations^35^. Further addition of glucose results in saturation and the catalytic current remains constant over time. In Fig. 16b, a plot of catalytic currents against varying glucose concentrations is given. As reported by Aftab et al.^36^, a linear calibration plot is obtained proving that the anodic peak current increases as a function of glucose concentration up to a saturation limit.

**Figure 17:**
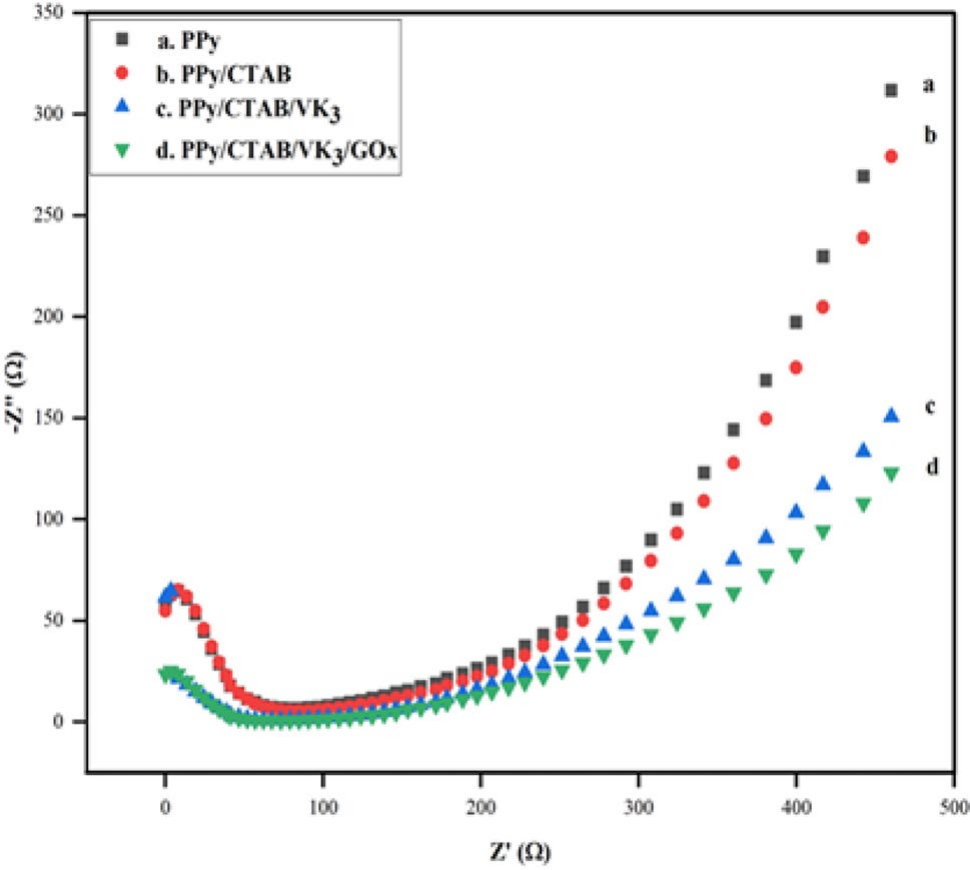
Nyquist plots of PPy, PPy/CTAB, PPy/CTAB/VK_3_, PPy/CTAB/VK_3_/GOx in 0.1 M K_4_Fe(CN)_6_ solution at a scan rate of 100 mV s^−1^.

The fabricated electrode surface was characterized by EIS to study the electronic transfer properties at the electrode–electrolyte interface. As shown in Fig. 17, the Nyquist plots displayed a semi-circle region of varying diameters followed by a linear region. The results of this spectroscopy include two distinct regions, first is the semi-circle region at larger frequencies, indicating the electron-transfer limited process; and the second is the straight-line region, at smaller frequencies, suggesting the diffusion-controlled process at the electrode–electrolyte interface. It is also evident from the results that the diffusion-controlled process of the bioanode PPy/CTAB/VK_3_/GOx is confirmed. Also, in curves (a,b,c) the smaller diameter of the semi-circle region affirms good electron transfer ability, whereas in (d) the larger diameter illustrates successful immobilization of VK_3_ and GOx on the bioanode.

## Conclusion

This study illustrates the successful hierarchical integration of a novel bioanode design PPy/CTAB/VK_3_/GOx generating a maximum current density of 6.35 mA cm^–2^ at 20 mM glucose concentration, close to the normal human physiological blood-glucose concentrations (10 mM). In this work, the effective conversion of metabolic energy to electric power in the cell results from the conversion of glucose to gluconolactone at the anode, which generates electrons. These electrons are shuttled by the redox mediator vitamin K_3,_ between the intensely hidden GOx active site and the electrode surface, coherently supported by the conductive filler of PPy. PPy owing to its semiconducting behavior, provided appropriate electrical communication at the electrode and also aided in the efficient immobilization of GOx. The results of this study indicated that the anode along with the high current output, also maintained its stability and performance over extended periods. This work advances the area of biocompatible electronics and presents a biofuel cell device that maintains an equilibrium between performance and environmental impact. Future work may involve utilizing carbon-based conducting supports including carbon dots, bucky paper, etc. for better biocatalytic performance. Further miniaturization of biofuel cells with higher power outputs and prolonged operational lifetimes for effective implantation in the human body may also be explored.

## Data Availability

The datasets used and/or analysed during the current study available from the corresponding author on reasonable request.
